# 4-(2-Benzoyl­ethyl)benzoic acid

**DOI:** 10.1107/S1600536808019387

**Published:** 2008-07-05

**Authors:** Roger A. Lalancette, Hugh W. Thompson

**Affiliations:** a Carl A. Olson Memorial Laboratories, Department of Chemistry, Rutgers University, Newark, NJ 07102, USA

## Abstract

The title compound, C_16_H_14_O_3_, adopts a conformation in which each functional group is almost coplanar with its adjacent ring, while the two aromatic rings are twisted with respect to one another with a dihedral angle of 78.51 (3)°. The compound dimerizes by standard centrosymmetric hydrogen-bonded carboxyl pairing [O⋯O = 2.6218 (11) Å and O—H⋯O = 176 (2)°]. The packing includes two inter­molecular C—H⋯O close contacts with the ketone group.

## Related literature

For related literature, see: Borthwick (1980 ▶); Steiner (1997 ▶).
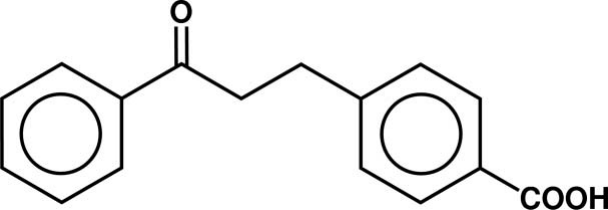


## Experimental

### 

#### Crystal data

C_16_H_14_O_3_
*M* *_r_* = 254.27
Monoclinic, 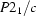

*a* = 7.3066 (1) Å
*b* = 8.7363 (1) Å
*c* = 19.6707 (3) Å
β = 90.296 (1)°
*V* = 1255.62 (3) Å^3^
*Z* = 4
Cu *K*α radiation
μ = 0.75 mm^−1^
*T* = 100 (2) K
0.27 × 0.21 × 0.15 mm

#### Data collection

Bruker SMART APEXII CCD area-detector diffractometer
Absorption correction: multi-scan (*SADABS*; Sheldrick, 2001 ▶) *T*_min_ = 0.871, *T*_max_ = 0.926
8836 measured reflections
2250 independent reflections
2122 reflections with *I* > 2σ(*I*)
*R*_int_ = 0.024

#### Refinement

*R*[*F* ^2^ > 2σ(*F* ^2^)] = 0.032
*wR*(*F* ^2^) = 0.085
*S* = 1.06
2250 reflections
177 parameters
H atoms treated by a mixture of independent and constrained refinement
Δρ_max_ = 0.24 e Å^−3^
Δρ_min_ = −0.15 e Å^−3^

### 

Data collection: *APEX2* (Bruker, 2006 ▶); cell refinement: *APEX2*; data reduction: *SAINT* (Bruker, 2005 ▶); program(s) used to solve structure: *SHELXTL* (Sheldrick, 2008 ▶); program(s) used to refine structure: *SHELXTL*; molecular graphics: *SHELXTL*; software used to prepare material for publication: *SHELXTL*.

## Supplementary Material

Crystal structure: contains datablocks I, global. DOI: 10.1107/S1600536808019387/fl2203sup1.cif

Structure factors: contains datablocks I. DOI: 10.1107/S1600536808019387/fl2203Isup2.hkl

Additional supplementary materials:  crystallographic information; 3D view; checkCIF report

## Figures and Tables

**Table 1:** Hydrogen-bond geometry (Å, °)

*D*—H⋯*A*	*D*—H	H⋯*A*	*D*⋯*A*	*D*—H⋯*A*
O3—H3*A*⋯O2^i^	0.97 (2)	1.66 (2)	2.6218 (11)	176 (2)
C5—H5⋯O1^ii^	0.95	2.59	3.3786 (14)	140
C8—H8*A*⋯O1^iii^	0.99	2.54	3.4864 (14)	160

Symmetry codes: (i) 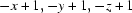; (ii) 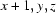; (iii) 
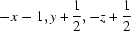
.

**Fig. 1.**
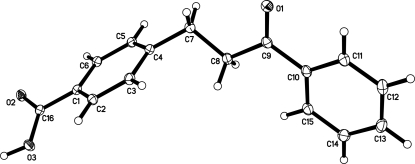
The asymmetric unit of (I), with its numbering. Displacement ellipsoids are drawn at the 30% probability level.

**Fig. 2.**
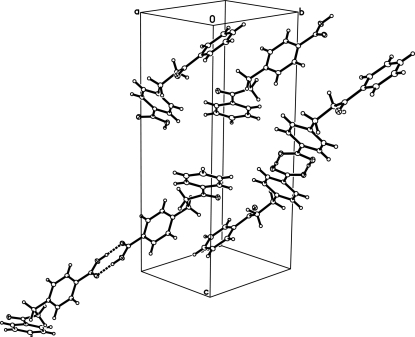
A partial packing diagram for (I), illustrating the dihedral relationships among the aromatic rings of the packed centrosymmetric dimers located at 1/2,1/2,1/2 and 1/2,0,0 in the unit cell. Displacement ellipsoids are drawn at the 30% probability level.

## supplementary crystallographic information

### Comment

Our X-ray study of ketocarboxylic acids seeks to uncover the structural features governing the choice among the five known keto–acid hydrogen-bonding modes. One major determinant is the availability of centrosymmetry, such that carboxyl dimerization, overall the commonest type of aggregation, is rare among single enantiomers. A second important influence is molecular flexibility, as reflected by the number of fully rotatable bonds in the molecule, with dimerization becoming less common as structural or conformational rigidity increases. In this context, the title molecule (I) is relatively flexible, containing four fully rotatable bonds in addition to the carboxyl.

Fig. 1 shows the asymmetric unit for (I) with its numbering. A principal feature is the near coplanarity of the two carbonyl-bearing functional groups with their respective benzene rings, permitting strong conjugation. The phenone's dihedral angle is 7.83 (8)° (C8—C9—C10—O1 *versus* C10—C11—C12—C13—C14—C15) and that for the carboxylic acid is 19.92 (6)° (C1—C16—O2—O3 *versus* C1—C2—C3—C4—C5—C6). The connecting alkyl chain, however, is not maximally staggered, the C4—C7—C8—C9 torsion being -160.20 (10)° rather than 180°. The two separate aromatic rings lie at a mutual dihedral angle of 78.51 (3)°.

Carboxyl dimers often display complete or partial averaging of C—O bond lengths and C—C—O angles due to disorder; however, no significant averaging is observed in (I), where these lengths and angles are similar to those in other highly ordered carboxyl situations (Borthwick, 1980).

Fig. 2 shows the packing arrangement for (I), typical for acids that are either racemic or, as with (I), achiral but capable of forming conformational racemates. Centrosymmetric dimers with two different orientations are centered at 1/2,1/2,1/2 and 1/2,0,0 in the chosen cell. The eight-membered carboxyl dimer of one orientational type lies close to the phenone aromatic ring in a dimer of the second type and nearly parallel to it [dihedral angle = 6.38 (7)°]. The normal distance from the centroid of this ring to the carboxyl-dimer plane = 3.472 Å and the intermolecular acid-to-ketone O═C···C═O distance = 3.3508 (15) Å.

Two intermolecular C—H···O═C close contacts were found in the packing, linking the ketone (O1) to H8A and to H5 in separate neighboring molecules (Table 1). These contacts lie within the 2.6 Å range we routinely survey for non-bonded dipolar packing interactions (Steiner, 1997).

### Experimental

Methyl 4-(3-oxo-3-phenyl-1-propenyl)benzoate, purchased from Acros Organics/Fisher Scientific, Springfield, NJ, USA, was hydrogenated in ethyl acetate at atmospheric pressure and room temperature over a 5% Pd/C catalyst. The resulting reduced methyl ester, m.p. *ca* 365 K, was saponified by refluxing with aqueous KOH to yield (I). Crystals of X-ray quality were obtained from Et_2_O, m.p. 431 K. Typically for carboxyl-paired keto acids, the solid-state (KBr) and the solution infrared spectra of (I) display only slight differences in the C═O region. The former features intense absorption at 1682 cm^-1^ for both C═O functions; in CHCl_3_ solution this combined peak is seen at 1688 cm^-1^.

### Refinement

All H atoms for (I) were found in electron density difference maps. The positional parameters and the isotropic thermal parameter of the O—H were allowed to refine fully. The methylene and the phenyl Hs were placed in geometrically idealized positions and constrained to ride on their parent C atoms with C—H distances of 0.99 Å for the methylene Hs and 0.95 Å for the phenyl Hs, and *U*_iso_(H) = 1.2*U*_eq_(C).

### Crystal data

**Table:**

C_16_H_14_O_3_	*F*_000_ = 536
*M**_r_* = 254.27	*D*_x_ = 1.345 Mg m^−^^3^
Monoclinic, *P*2_1_/*c*	Melting point: 431 K
Hall symbol: -P 2ybc	Cu *K*α radiation λ = 1.54178 Å
*a* = 7.3066 (1) Å	Cell parameters from 6862 reflections
*b* = 8.7363 (1) Å	θ = 5.1–69.6º
*c* = 19.6707 (3) Å	µ = 0.75 mm^−^^1^
β = 90.296 (1)º	*T* = 100 (2) K
*V* = 1255.62 (3) Å^3^	Block, colourless
*Z* = 4	0.27 × 0.21 × 0.15 mm

### Data collection

**Table:**

Bruker SMART APEXII CCD area-detector diffractometer	2250 independent reflections
Radiation source: fine-focus sealed tube	2122 reflections with *I* > 2σ(*I*)
Monochromator: graphite	*R*_int_ = 0.024
*T* = 100(2) K	θ_max_ = 69.9º
φ and ω scans	θ_min_ = 5.5º
Absorption correction: multi-scan(SADABS; Sheldrick, 2001)	*h* = −8→7
*T*_min_ = 0.871, *T*_max_ = 0.927	*k* = −10→10
8836 measured reflections	*l* = −20→23

### Refinement

**Table:**

Refinement on *F*^2^	Hydrogen site location: inferred from neighbouring sites
Least-squares matrix: full	H atoms treated by a mixture of independent and constrained refinement
*R*[*F*^2^ > 2σ(*F*^2^)] = 0.032	*w* = 1/[σ^2^(*F*_o_^2^) + (0.0362*P*)^2^ + 0.5203*P*] where *P* = (*F*_o_^2^ + 2*F*_c_^2^)/3
*wR*(*F*^2^) = 0.085	(Δ/σ)_max_ < 0.001
*S* = 1.06	Δρ_max_ = 0.24 e Å^−^^3^
2250 reflections	Δρ_min_ = −0.15 e Å^−^^3^
177 parameters	Extinction correction: SHELXTL (Sheldrick, 2008), Fc^*^=kFc[1+0.001xFc^2^λ^3^/sin(2θ)]^-1/4^
Primary atom site location: structure-invariant direct methods	Extinction coefficient: 0.0010 (3)
Secondary atom site location: difference Fourier map	

### Special details

**Table:**

Experimental. 'crystal mounted on cryoloop using Paratone-N'
Geometry. All e.s.d.'s (except the e.s.d. in the dihedral angle between two l.s. planes) are estimated using the full covariance matrix. The cell e.s.d.'s are taken into account individually in the estimation of e.s.d.'s in distances, angles and torsion angles; correlations between e.s.d.'s in cell parameters are only used when they are defined by crystal symmetry. An approximate (isotropic) treatment of cell e.s.d.'s is used for estimating e.s.d.'s involving l.s. planes.
Refinement. Refinement of *F*^2^ against ALL reflections. The weighted *R*-factor *wR* and goodness of fit *S* are based on *F*^2^, conventional *R*-factors *R* are based on *F*, with *F* set to zero for negative *F*^2^. The threshold expression of *F*^2^ > σ(*F*^2^) is used only for calculating *R*-factors(gt) *etc*. and is not relevant to the choice of reflections for refinement. *R*-factors based on *F*^2^ are statistically about twice as large as those based on *F*, and *R*- factors based on ALL data will be even larger.

### Fractional atomic coordinates and isotropic or equivalent isotropic displacement parameters (Å^2^)

**Table:**

	*x*	*y*	*z*	*U*_iso_*/*U*_eq_	
O1	−0.51626 (11)	0.00590 (9)	0.20479 (4)	0.0244 (2)	
C1	0.20287 (16)	0.31711 (13)	0.39461 (6)	0.0183 (2)	
O2	0.49306 (11)	0.35014 (10)	0.44785 (4)	0.0225 (2)	
C2	0.01509 (16)	0.34231 (13)	0.40114 (6)	0.0201 (3)	
H2	−0.0289	0.4121	0.4343	0.024*	
C3	−0.10693 (16)	0.26552 (14)	0.35930 (6)	0.0209 (3)	
H3	−0.2345	0.2828	0.3643	0.025*	
O3	0.27242 (12)	0.52180 (10)	0.46838 (4)	0.0237 (2)	
H3A	0.363 (3)	0.565 (3)	0.4988 (12)	0.078 (7)*	
C4	−0.04556 (16)	0.16284 (13)	0.30981 (6)	0.0192 (3)	
C5	0.14214 (16)	0.13923 (13)	0.30362 (6)	0.0198 (3)	
H5	0.1863	0.0702	0.2702	0.024*	
C6	0.26570 (16)	0.21514 (13)	0.34560 (6)	0.0197 (3)	
H6	0.3933	0.1975	0.3409	0.024*	
C7	−0.18159 (16)	0.08144 (13)	0.26458 (6)	0.0215 (3)	
H7A	−0.2732	0.0288	0.2932	0.026*	
H7B	−0.1170	0.0028	0.2376	0.026*	
C8	−0.27993 (16)	0.19232 (14)	0.21631 (6)	0.0214 (3)	
H8A	−0.3053	0.2887	0.2410	0.026*	
H8B	−0.1973	0.2171	0.1781	0.026*	
C9	−0.45757 (16)	0.13083 (13)	0.18776 (6)	0.0199 (3)	
C10	−0.56580 (16)	0.23037 (14)	0.14032 (6)	0.0206 (3)	
C11	−0.74205 (17)	0.18477 (15)	0.12200 (6)	0.0238 (3)	
H11	−0.7893	0.0907	0.1386	0.029*	
C12	−0.84914 (18)	0.27529 (16)	0.07976 (6)	0.0279 (3)	
H12	−0.9689	0.2432	0.0673	0.033*	
C13	−0.78050 (18)	0.41321 (16)	0.05569 (6)	0.0290 (3)	
H13	−0.8538	0.4756	0.0268	0.035*	
C14	−0.60602 (18)	0.46002 (15)	0.07354 (6)	0.0272 (3)	
H14	−0.5598	0.5546	0.0571	0.033*	
C15	−0.49839 (17)	0.36867 (14)	0.11544 (6)	0.0232 (3)	
H15	−0.3781	0.4006	0.1272	0.028*	
C16	0.33506 (15)	0.39769 (13)	0.43939 (5)	0.0184 (3)	

### Atomic displacement parameters (Å^2^)

**Table:**

	*U*^11^	*U*^22^	*U*^33^	*U*^12^	*U*^13^	*U*^23^
O1	0.0238 (5)	0.0218 (4)	0.0277 (5)	−0.0025 (3)	−0.0032 (3)	0.0005 (3)
C1	0.0207 (6)	0.0181 (5)	0.0162 (5)	0.0004 (4)	−0.0022 (4)	0.0026 (4)
O2	0.0187 (5)	0.0262 (5)	0.0224 (4)	0.0019 (3)	−0.0038 (3)	−0.0036 (3)
C2	0.0225 (6)	0.0205 (6)	0.0172 (6)	0.0024 (4)	0.0000 (4)	−0.0012 (4)
C3	0.0176 (6)	0.0234 (6)	0.0216 (6)	0.0010 (4)	−0.0012 (4)	−0.0001 (5)
O3	0.0227 (5)	0.0231 (4)	0.0254 (4)	0.0016 (3)	−0.0040 (3)	−0.0072 (3)
C4	0.0223 (6)	0.0184 (6)	0.0169 (6)	−0.0008 (4)	−0.0031 (4)	0.0019 (4)
C5	0.0238 (6)	0.0189 (6)	0.0168 (5)	0.0024 (4)	−0.0006 (4)	−0.0007 (4)
C6	0.0185 (6)	0.0213 (6)	0.0192 (6)	0.0019 (4)	−0.0012 (4)	0.0013 (4)
C7	0.0224 (6)	0.0206 (6)	0.0214 (6)	0.0000 (5)	−0.0034 (4)	−0.0025 (5)
C8	0.0219 (6)	0.0223 (6)	0.0200 (6)	−0.0021 (5)	−0.0031 (4)	−0.0005 (5)
C9	0.0207 (6)	0.0213 (6)	0.0177 (6)	0.0004 (4)	0.0011 (4)	−0.0045 (4)
C10	0.0223 (6)	0.0237 (6)	0.0157 (5)	0.0006 (5)	−0.0007 (4)	−0.0045 (5)
C11	0.0249 (7)	0.0267 (6)	0.0199 (6)	−0.0022 (5)	−0.0017 (5)	−0.0023 (5)
C12	0.0243 (7)	0.0366 (7)	0.0228 (6)	0.0008 (5)	−0.0054 (5)	−0.0038 (5)
C13	0.0334 (7)	0.0332 (7)	0.0205 (6)	0.0073 (6)	−0.0068 (5)	−0.0006 (5)
C14	0.0359 (7)	0.0248 (6)	0.0210 (6)	−0.0002 (5)	−0.0018 (5)	0.0009 (5)
C15	0.0252 (7)	0.0249 (6)	0.0196 (6)	−0.0014 (5)	−0.0023 (5)	−0.0029 (5)
C16	0.0200 (6)	0.0198 (6)	0.0153 (5)	0.0001 (4)	0.0000 (4)	0.0021 (4)

### Geometric parameters (Å, °)

**Table:**

O1—C9	1.2202 (14)	C7—H7A	0.9900
C1—C6	1.3923 (16)	C7—H7B	0.9900
C1—C2	1.3961 (17)	C8—C9	1.5104 (16)
C1—C16	1.4818 (15)	C8—H8A	0.9900
O2—C16	1.2373 (14)	C8—H8B	0.9900
C2—C3	1.3837 (16)	C9—C10	1.4983 (16)
C2—H2	0.9500	C10—C11	1.3936 (17)
C3—C4	1.3992 (16)	C10—C15	1.3944 (17)
C3—H3	0.9500	C11—C12	1.3861 (18)
O3—C16	1.3088 (14)	C11—H11	0.9500
O3—H3A	0.97 (2)	C12—C13	1.389 (2)
C4—C5	1.3928 (17)	C12—H12	0.9500
C4—C7	1.5087 (15)	C13—C14	1.3825 (19)
C5—C6	1.3889 (16)	C13—H13	0.9500
C5—H5	0.9500	C14—C15	1.3885 (17)
C6—H6	0.9500	C14—H14	0.9500
C7—C8	1.5326 (16)	C15—H15	0.9500
			
C6—C1—C2	119.49 (11)	C9—C8—H8B	108.8
C6—C1—C16	119.96 (10)	C7—C8—H8B	108.8
C2—C1—C16	120.55 (10)	H8A—C8—H8B	107.7
C3—C2—C1	119.95 (10)	O1—C9—C10	120.34 (11)
C3—C2—H2	120.0	O1—C9—C8	121.24 (11)
C1—C2—H2	120.0	C10—C9—C8	118.36 (10)
C2—C3—C4	121.12 (11)	C11—C10—C15	118.98 (11)
C2—C3—H3	119.4	C11—C10—C9	118.63 (11)
C4—C3—H3	119.4	C15—C10—C9	122.36 (11)
C16—O3—H3A	110.8 (13)	C12—C11—C10	120.66 (12)
C5—C4—C3	118.36 (10)	C12—C11—H11	119.7
C5—C4—C7	121.64 (10)	C10—C11—H11	119.7
C3—C4—C7	120.00 (10)	C11—C12—C13	119.70 (12)
C6—C5—C4	120.97 (11)	C11—C12—H12	120.1
C6—C5—H5	119.5	C13—C12—H12	120.1
C4—C5—H5	119.5	C14—C13—C12	120.27 (12)
C5—C6—C1	120.11 (11)	C14—C13—H13	119.9
C5—C6—H6	119.9	C12—C13—H13	119.9
C1—C6—H6	119.9	C13—C14—C15	119.96 (12)
C4—C7—C8	111.89 (9)	C13—C14—H14	120.0
C4—C7—H7A	109.2	C15—C14—H14	120.0
C8—C7—H7A	109.2	C14—C15—C10	120.42 (12)
C4—C7—H7B	109.2	C14—C15—H15	119.8
C8—C7—H7B	109.2	C10—C15—H15	119.8
H7A—C7—H7B	107.9	O2—C16—O3	123.21 (10)
C9—C8—C7	113.88 (10)	O2—C16—C1	121.69 (10)
C9—C8—H8A	108.8	O3—C16—C1	115.10 (10)
C7—C8—H8A	108.8		
			
C6—C1—C2—C3	0.34 (17)	C8—C9—C10—C11	−170.32 (10)
C16—C1—C2—C3	−179.56 (10)	O1—C9—C10—C15	−175.08 (11)
C1—C2—C3—C4	−0.39 (17)	C8—C9—C10—C15	7.87 (16)
C2—C3—C4—C5	0.11 (17)	C15—C10—C11—C12	−0.05 (18)
C2—C3—C4—C7	−179.60 (10)	C9—C10—C11—C12	178.20 (10)
C3—C4—C5—C6	0.22 (17)	C10—C11—C12—C13	−0.32 (18)
C7—C4—C5—C6	179.93 (10)	C11—C12—C13—C14	0.20 (19)
C4—C5—C6—C1	−0.27 (17)	C12—C13—C14—C15	0.29 (19)
C2—C1—C6—C5	−0.01 (17)	C13—C14—C15—C10	−0.67 (18)
C16—C1—C6—C5	179.89 (10)	C11—C10—C15—C14	0.55 (17)
C5—C4—C7—C8	−112.51 (12)	C9—C10—C15—C14	−177.64 (10)
C3—C4—C7—C8	67.19 (14)	C6—C1—C16—O2	−19.53 (16)
C4—C7—C8—C9	−160.20 (10)	C2—C1—C16—O2	160.37 (11)
C7—C8—C9—O1	2.55 (16)	C6—C1—C16—O3	160.00 (10)
C7—C8—C9—C10	179.57 (9)	C2—C1—C16—O3	−20.10 (15)
O1—C9—C10—C11	6.72 (16)		

### Hydrogen-bond geometry (Å, °)

**Table:**

*D*—H···*A*	*D*—H	H···*A*	*D*···*A*	*D*—H···*A*
O3—H3A···O2^i^	0.97 (2)	1.66 (2)	2.6218 (11)	176 (2)
C5—H5···O1^ii^	0.95	2.59	3.3786 (14)	140
C8—H8A···O1^iii^	0.99	2.54	3.4864 (14)	160

Symmetry codes: (i) −*x*+1, −*y*+1, −*z*+1; (ii) *x*+1, *y*, *z*; (iii) −*x*−1, *y*+1/2, −*z*+1/2.
